# The First Evidence of *Cryptosporidium meleagridis* Infection in a Colon Adenocarcinoma From an Immunocompetent Patient

**DOI:** 10.3389/fcimb.2019.00035

**Published:** 2019-03-04

**Authors:** Żaneta Kopacz, Martin Kváč, Paweł Karpiński, Andrzej B. Hendrich, Maria M. Sąsiadek, Przemysław Leszczyński, Bohumil Sak, John McEvoy, Marta Kicia

**Affiliations:** ^1^Department of Biology and Medical Parasitology, Wrocław Medical University, Wrocław, Poland; ^2^Institute of Parasitology, Biology Centre of the Czech Academy of Sciences, České Budějovice, Czechia; ^3^Faculty of Agriculture, University of South Bohemia, České Budějovice, Czechia; ^4^Department of Genetics, Wrocław Medical University, Wrocław, Poland; ^5^Department of Microbiological Sciences, North Dakota State University, Fargo, ND, United States

**Keywords:** *Cryptosporidium meleagridis*, colon cancer, adenocarcinoma, colon infection, PCR, immunofluorescence labeling

## Abstract

**Objectives:** The potential linkage between *Cryptosporidium* spp. infection and colorectal human cancer was suggested by limited reports showing higher prevalence of *C. parvum* and *C. hominis* in patients with colon cancer. Here we conducted research concerning presence of *Cryptosporidium* spp. in malignant tissue collected from patients with colorectal cancer.

**Methods:** Cancerous colon tissue samples collected from 145 non-HIV infected patients with colorectal cancer were screened for *Cryptosporidium* spp. by immunofluorescence antibody test and genus-specific nested polymerase chain reaction followed by sequencing.

**Results:** Screened pathogen was found in cancerous tissue originating from immunocompetent man with colon adenocarcinoma. Genotyping revealed presence of *Cryptosporidium meleagridis*. The presence of *Cryptosporidium* life cycle stages (oocysts and endogenous stages) in colon carcinoma tissue was confirmed by genus-specific FITC-labeling.

**Conclusions:** Herein, we report on a *C. meleagridis* infection of a colon adenocarcinoma in an immunocompetent patient. This is the first report of *C. meleagridis* infection in the human colon and first evidence of active development of this species in cancer tissue.

## Introduction

Cryptosporidiosis, a diarrheal disease caused by species of protist parasites in the genus *Cryptosporidium*, can be severe and life-threatening in those with a compromised or underdeveloped immune system. More than 90% of human cases are caused by *C. hominis*, a species that is generally restricted to humans, and *C. parvum*, a species that infects a broad range of mammals. *Cryptosporidium meleagridis*, a species that primarily infects birds, but which also infects various mammal species, is the third most common cause of cryptosporidiosis in humans (Xiao, 2010). Estimated prevalence of human cryptosporidiosis in Poland (1.4–2.3%) has been available from limited research studies and based on the data from the National Institute of Public Health, National Institute of Hygiene in Poland and 24 findings of *Cryptosporidium* spp. have been reported from 2007 to 2016 (Wolska-Kusnierz et al., 2007; Bajer et al., 2008; Wesołowska et al., 2016; Bednarska et al., 2018). The majority of infection cases was caused by *C. parvum* and *C. hominis*. To date, *C. felis* infection has been detected in a child after liver transplantation (Bednarska et al., 2018) and *C. meleagridis* infection has only been confirmed in three immunodeficient patients; two children, with CD40L primary deficiency (Bajer et al., 2008) and with X linked hyper-IgM syndrome type 1 (XHIM syndrome) (Wolska-Kusnierz et al., 2007), and in a woman with AIDS suffering from persisted diarrhea (Wesołowska et al., 2016). Human infection by *C. meleagridis* is more frequent in some populations in Thailand or Peru. In the rest of the world, such cases have been linked to travels to endemic countries or to contact with poultry (Leoni et al., 2006; Cama et al., 2008; Elwin et al., 2012; Silverlås et al., 2012).

Worldwide, colorectal cancer is the second most frequently diagnosed cancer in females and the third in males. Major risk factors for colorectal cancer include age, personal or family history of chronic inflammatory bowel disease, gene mutations, high consumption of red or processed meat, smoking, physical inactivity, obesity and moderate to heavy alcohol consumption (Subramaniam et al., 2016). Other risk factors include bacterial, viral and parasitic infections (van Tong et al., 2017).

Epidemiological studies have shown an association between colorectal cancer and *Cryptosporidium* spp. infection (Osman et al., 2018; Sulzyc-Bielicka et al., 2018). Furthermore, a limited number of experimental studies have shown that *C. parvum* induces neoplastic changes in immunocompromised animals and in cells cultured *in vitro*, consistent with a role in carcinogenesis (Benamrouz et al., 2012a,b).

The present study is aimed to determine the presence of *Cryptosporidium* spp. infection in tissue samples from colorectal tumors. We describe an active *C. meleagridis* infection in samples from malignant tissue of non-HIV infected immunocompetent male patient with colon adenocarcinoma. To our knowledge, this is the first report of the case of *C. meleagridis* infection in the colon of a human.

## Patients and Methods

Samples of colic neoplasia obtained during colectomies of 145 patients, who were being treated for colorectal cancer at the First Department of Surgical Oncology, Lower Silesian Oncology Center in Wrocław (Poland) between 2009 and 2010 were screened for *Cryptosporidium* infection between 2017 and 2018. The inclusion criteria for of all of the patients in this study were a) primary sporadic CRC (no history of hereditary/familial CRC), (b) HIV negativity, (c) not receiving an immunosuppressive treatment, (d) no chemotherapy and/or radiotherapy before surgical resection.

Tissues of colorectal cancer from all patients were screened for the presence of specific DNA of *Cryptosporidium* spp. using molecular methods and presence of *Cryptosporidium* developmental stages using the immunofluorescence antibody test (IFA). Samples were collected intraoperatively and aseptically. All tissue samples were stored in RNAlater™ Stabilization Solution (Thermo Fisher Scientific, Carlsbad, CA, United States) and immediately frozen at −70°C. A total of 200 mg of tissue was homogenized by bead disruption for 60 s at 5.5 m/s with 0.5 mm glass beads using a Precellys 24 Instrument (Bertin Technologies, France). Genomic DNA was subsequently isolated using the Gentra Puregene Tissue Kit (Qiagen, Hilden, Germany) according to the manufacturer's instructions. DNA quality was verified by NanoDrop (Thermo Fisher Scientific, Carlsbad, CA, United States) measurements and β-globin gene amplification (Pan et al., 2001). To detect *Cryptosporidium* specific DNA, a nested protocol was used to amplify a partial region of the small ribosomal subunit rRNA (SSU; ~830 bp) gene (Xiao et al., 1999; Jiang et al., 2005). Only samples that were positive for SSU were screened for the 60 kDa glycoprotein (gp60; ~900 bp) gene (Glaberman et al., 2001; Alves et al., 2003). The PCR conditions were as previously described (Xiao et al., 1999; Glaberman et al., 2001). Negative (molecular grade water) and positive (DNA of *C. proliferans*) controls were included with each PCR amplification. Secondary products were purified (QIAquick^®;^ Gel Extraction Kit, Qiagen, Hilden, Germany) and sequencing was carried out in both directions using an ABI 3130 sequencer (Applied Biosystems, Foster City, CA, United States). Amplification and sequencing of each locus were repeated three times. The nucleotide sequences in this study were manually edited using the program ChromasPro 2.1.4 (Technelysium, Pty, Ltd., South Brisbane, Australia) and aligned with previously published sequences. Sequences of SSU and gp60 derived in this study have been deposited in GenBank under accession numbers MK311181 and MK311182.

For IFA analysis, tissue samples were thawed at laboratory temperature, mechanically homogenized using a sterile mortar and pestle, and smeared on sterile slides (ten for each labeling). The presence of *Cryptosporidium* oocysts was examined using differential interference contrast (DIC) and fluorescence microscopy following labeling with a fluorescein-tagged mouse monoclonal antibody that is specific for the *Cryptosporidium* oocyst wall (*Cryptosporidium* IF Test, Crypto cel, Cellabs Pty Ltd., Brookvale, Australia). Endogenous life stages were examined under DIC and fluorescence microscopy following labeling with fluorescein-tagged rat anti-*Cryptosporidium parvum* sporozoite polyclonal antibody, which is specific for sporozoites, merozoites, and all other intracellular reproductive stages (A600FLR-20X Sporo-Glo™, Waterborne, INC. New Orleans, LA, United States).

This study was approved by the Human Research Ethics Committee of Wrocław Medical University (agreement number KB-328/2009). Written informed consent was obtained from every participant prior to examination.

## Results

Among all patients (*n* = 145) the mean age was 65 ± 10.2 years, and ranged between 35–88 years. Overall, the male-to-female ratio was 77 (53%) to 68 (47%). The study group included 34 patients with proximal (right part of colon inclusive of transverse colon) and 111 patients with distal (left part of colon) tumor location. *Cryptosporidium meleagridis* DNA was confirmed in one patient and no other species of *Cryptosporidium* were found in the tumor tissues of the rest of study group.

*Cryptosporidium-*positive patient was a 71-year-old HIV-negative man who presented with a 1-year history of bloody stools, increased difficulty with defecation, abdominal pain, flatulence, increased fatigue and unintended weight loss. He had a 20-year history of regular smoking above 2 packs of cigarettes per week, seldomly drank alcohol (median 50 g of pure alcohol per week), and had no previous medical or surgical history of note. Additionally, he had no risk factors for, or blood result anomalies suggestive of, underlying immunodeficiency. A colonoscopy identified a lesion (pre-cancerous polyp) located 20 cm from ileocecal valve. Histopathological examination of biopsy tissue showed the presence of a stage IIA adenocarcinoma, according to the TNM Classification of Malignant Tumors (Tumor, Nodes, Metastasis, TNM) of staging as maintained by the American Joint Committee on Cancer (AJCC) and Union for International Cancer Control (UICC) (Hari et al., 2013). The patient was admitted to the hospital to undergo a planned right hemicolectomy of an ascending adenocarcinoma.

**Figure 1:**
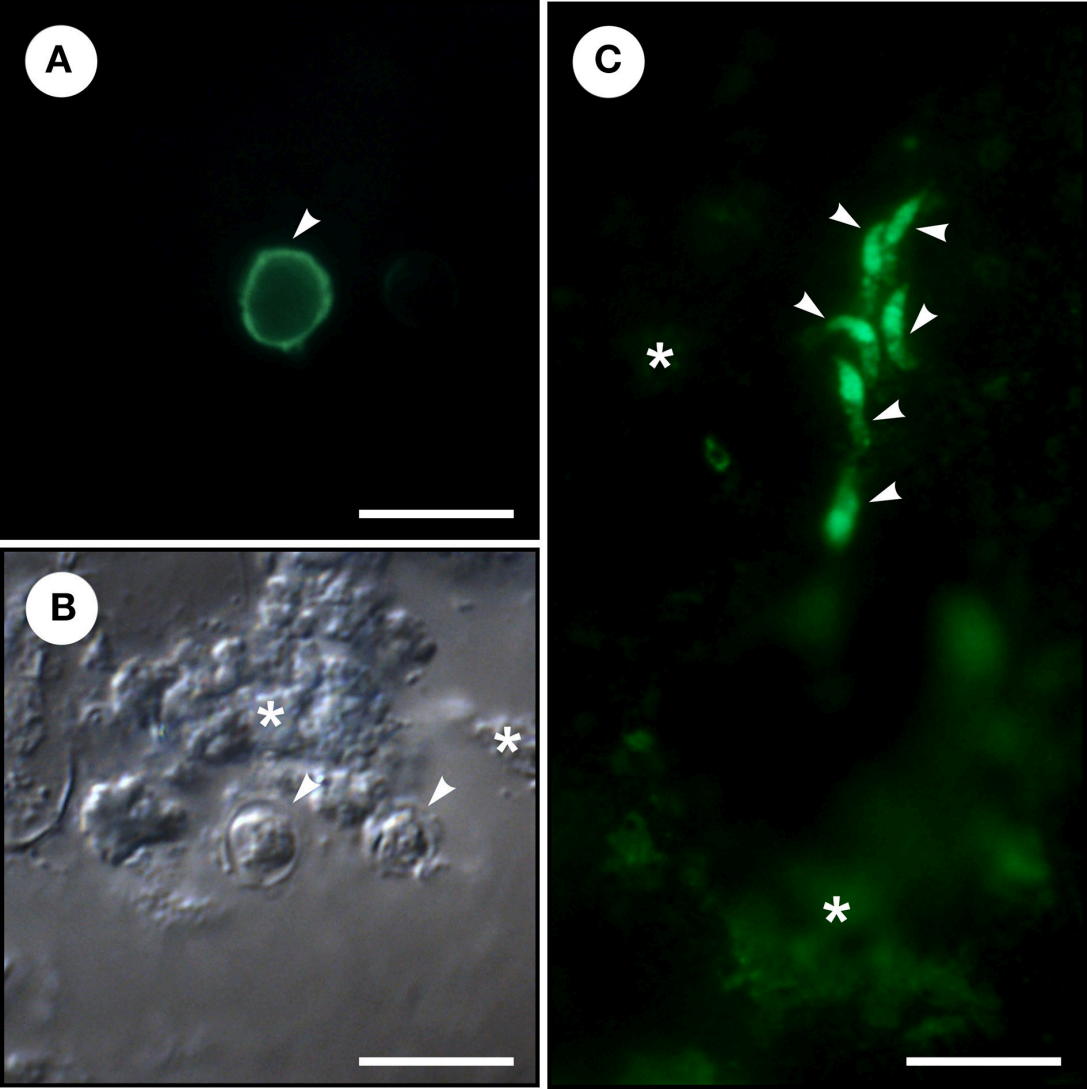
Microscopic examination of homogenized colonical tissue from a patient with adenocarcinoma and *Cryptosporidium meleagridis* infection. **(A)** *Cryptosporidium* oocyst (arrowhead) labeled with fluorescein-labeled mouse monoclonal antibody binds to *Cryptosporidium* oocyst wall (*Cryptosporidium* IF Test, Crypto cel, Cellabs Pty Ltd., Brookvale, Australia). **(B)** *Cryptosporidium* oocysts (arrowheads) under differential interference contrast and colonical tissue (asterisks). **(C)** Six merozoites (arrowheads) labeled with fluorescein-labeled rat anti-*Cryptosporidium parvum* sporozoite polyclonal antibody binds to sporozoites, merozoites, and all other intracellular reproductive stages (A600FLR-20X Sporo-Glo™, Waterborne, INC. New Orleans, LA, USA) and colonical tissue (asterisks). Bar = 10 μm.

The presence of *Cryptosporidium* life cycle stages (oocysts and endogenous stages) in colon carcinoma tissue was confirmed by genus-specific FITC-labeling (Figure 1). Phylogenetic analyses of SSU rRNA sequences revealed the presence of *C. meleagridis* that was identical to isolates previously reported in humans and birds (GenBank acc. Nos. AY166839 and KY352486). Subtyping of *C. meleagridis* isolates at the gp60 locus showed the presence of subtype family IIIg previously reported in a human from Nepal and Sweden (GenBank acc. Nos. KU852730 and KJ210619) and birds from Algeria (GenBank acc. Nos. KY352480, KY352481, JX878610).

## Discussion

In this study, microscopic methods have demonstrated the presence of *C. meleagridis* endogenous stages (merozoites) in adenocarcinoma tissue in the colon. To our knowledge this is the first report of *C. meleagridis* in the human colon, and the identification of merozoites indicates an active infection. In the only other study to date in humans, *C. meleagridis* infection was localized to the ileocaecal valve, a sphincter muscle that separates the small and large intestine, in a person who was immunocompromised following a hematopoietic stem cell transplantation (Kagawa et al., 2018). Minor *C. meleagridis* infection has been reported in the colon of birds (chickens and turkeys) and mice; however, the infection intensity was low relative to that in the small intestine, which was the major site of infection (Pavlásek, 1994; Akiyoshi et al., 2003). Several recent studies have shown an increased prevalence of *Cryptosporidium* in patients with intestinal cancers. For example, in Poland, *Cryptosporidium* coproantigen was significantly more prevalent in patients with colorectal cancer than in a control group without malignant changes (Sulzyc-Bielicka et al., 2018). Similarly, Osman et al. (2018) showed that DNA and oocysts of *C. parvum* and *C. hominis* were more prevalent in digestive biopsies of patients with diagnosed colon neoplasia/adenocarcinoma compared to patients without digestive neoplasia. Moreover, according to Berahmat et al. (2017) the rate of cryptosporidiosis in children with cancer undergoing chemotherapy was higher (3.8%) than the one in the control group (0%). Although, while using an immunosuppressed mouse model, chronic *C. parvum* infection has been reported to cause neoplastic changes in the small intestine, suggesting a potential role as a carcinogen (Benamrouz et al., 2012a,b), the contribution of *Cryptosporidium* spp. to cancer etiology has been largely unexplored (Cheeseman et al., 2016). Taking into account the fact that among 145 examined patients in our study only one was infected with *C. meleagridis*, we consider this infection as opportunistic. Considering that human adenocarcinoma usually takes above 10 years to be diagnosed (Subramaniam et al., 2016), it is more likely that the *C. meleagridis* infection in the present study has resulted from the tumor presence. Our patient infected with *C. meleagridis* subtype IIIg, has not reported direct contact with poultry and wild birds, and the source of infection remains unknown. Nevertheless, the occurrence of this subtype as well as the other *C. meleagridis* subtypes (IIIb, IIIc, IIIe, IIIf, IIIh and IIIi) in humans, indicates that they are susceptible to almost all subtypes of *C. meleagridis* (Abal-Fabeiro et al., 2013; Wang et al., 2013). Future studies should examine characteristics of carcinoma tissue that could increase susceptibility to *Cryptosporidium* infection, including altered mucin expression and loss of epithelial cell integrity (Nath and Mukherjee, 2014; Cheeseman et al., 2016).

Although our results have shown that *C. meleagridis* might inhabit pathologically changed tumor tissue in human colon, there is still no evidence that this apicomplexan parasite should be considered as carcinogenic agent to humans according to International Agency for Research on Cancer (IARC).

## Author Contributions

ŻK and MKi designed research. ŻK, MKv, PK, PL, BS, and MKi performed research. ŻK, MKv, PK, AH, MS, PL, JM, and MKi analyzed data. ŻK, MKv, JM, and MKi wrote the manuscript. AH and MS revised the manuscript for important intellectual content.

### Conflict of Interest Statement

The authors declare that the research was conducted in the absence of any commercial or financial relationships that could be construed as a potential conflict of interest.

## References

[B1] Abal-Fabeiro J. L. Maside X. Bello X. Llovo J. Bartolomé C. (2013). Multilocus patterns of genetic variation across *Cryptosporidium* species suggest balancing selection at the *gp60* locus. Mol. Ecol. 22, 4723–4732. 10.1111/mec.12425 23915002

[B2] Akiyoshi D. E. Dilo J. Pearson C. Chapman S. Tumwine J. Tzipori S. (2003). Characterization of *Cryptosporidium meleagridis* of human origin passaged through different host species. Infect. Immun. 71, 1828–1832. 10.1128/IAI.71.4.1828-1832.2003 12654797 PMC152090

[B3] Alves M. Xiao L. Sulaiman I. Lal A. A. Matos O. Antunes F. (2003). Subgenotype analysis of *Cryptosporidium* isolates from humans, cattle, and zoo ruminants in Portugal. J. Clin. Microbiol. 41, 2744–2747. 10.1128/JCM.41.6.2744-2747.2003 12791920 PMC156540

[B4] Bajer A. Bednarska M. Cacciò S. M. Wolska-Kuśnierz B. Heropolitanska-Pliszka E. Bernatowska E.. (2008). Genotyping of *Cryptosporidium* isolates from human clinical cases in Poland. Parasitol. Res. 103, 37–42. 10.1007/s00436-008-0924-5 18301922

[B5] Bednarska M. Jankowska I. Pawelas A. Piwczynska K. Bajer A. Wolska-Kuśnierz B.. (2018). Prevalence of *Cryptosporidium, Blastocystis*, and other opportunistic infections in patients with primary and acquired immunodeficiency. Parasitol. Res. 117, 2869–2879. 10.1007/s00436-018-5976-6 29946765 PMC6105259

[B6] Benamrouz S. Conseil V. Creusy C. Calderon E. Dei-cas E. Certad G. (2012a). Parasites and malignancies, a review, with emphasis on digestive cancer induced by *Cryptosporidium parvum* (Alveolata: Apicomplexa). Parasite 19, 101–115. 10.1051/parasite/2012192101 22348213 PMC3671432

[B7] Benamrouz S. Guyot K. Gazzola S. Mouray A. Chassat T. Delaire B.. (2012b). *Cryptosporidium parvum* infection in SCID mice infected with only one oocyst: qPCR assessment of parasite replication in tissues and development of digestive cancer. PLoS ONE 7:e51232. 10.1371/journal.pone.0051232 23272093 PMC3521773

[B8] Berahmat R. Mahami-Oskouei M. Rezamand A. Spotin A. Aminisani N. Ghoyounchi R.. (2017). *Cryptosporidium* infection in children with cancer undergoing chemotherapy: how important is the prevention of opportunistic parasitic infections in patients with malignancies? Parasitol. Res. 116, 2507–2515. 10.1007/s00436-017-5560-5 28730516

[B9] Cama V. A. Bern C. Roberts J. Cabrera L. Sterling C. R. Ortega Y.. (2008). *Cryptosporidium* species and subtypes and clinical manifestations in children, Peru. Emerg. Infect. Dis. 14, 1567–1574. 10.3201/eid1410.071273 18826821 PMC2609889

[B10] Cheeseman K. Certad G. Weitzman J. B. (2016). [Parasites and cancer: is there a causal link?]. Med. Sci. 32, 867–873. 10.1051/medsci/20163210020 27758751

[B11] Elwin K. Hadfield S. J. Robinson G. Chalmers R. M. (2012). The epidemiology of sporadic human infections with unusual cryptosporidia detected during routine typing in England and Wales, 2000–2008. Epidemiol. Infect. 140, 673–683. 10.1017/S0950268811000860 21733255

[B12] Glaberman S. Sulaiman I. M. Bern C. Limor J. Peng M. M. Morgan U.. (2001). A multilocus genotypic analysis of *Cryptosporidium meleagridis*. J. Eukaryot. Microbiol. (Suppl.), 19S−22S. 10.1111/j.1550-7408.2001.tb00439.x 11906063

[B13] Hari D. M. Leung A. M. Lee J.-H. Sim M.-S. Vuong B. Chiu C. G.. (2013). AJCC cancer staging manual 7th edition criteria for colon cancer: do the complex modifications improve prognostic assessment? J. Am. Coll. Surg. 217, 181–190. 10.1016/j.jamcollsurg.2013.04.018 23768788 PMC4657944

[B14] Jiang J. Alderisio K. A. Xiao L. (2005). Distribution of *Cryptosporidium* genotypes in storm event water samples from three watersheds in New York. Appl. Environ. Microbiol. 71, 4446–4454. 10.1128/AEM.71.8.4446-4454.2005 16085835 PMC1183313

[B15] Kagawa K. Fujino H. Miki H. Sogabe K. Takahashi M. Maruhashi T.. (2018). Cryptosporidiosis in a transplant recipient with severe intractable diarrhea: detection of *Cryptosporidium* oocysts by intestinal biopsies. Transpl. Infect. Dis. 20, e12826. 10.1111/tid.12826 29277954

[B16] Leoni F. Amar C. Nichols G. Pedraza-Díaz S. McLauchlin J. (2006). Genetic analysis of *Cryptosporidium* from 2414 humans with diarrhoea in England between 1985 and 2000. J. Med. Microbiol. 55, 703–707. 10.1099/jmm.0.46251-0 16687587

[B17] Nath S. Mukherjee P. (2014). MUC1: A multifaceted oncoprotein with a key role in cancer progression. Trends Mol. Med. 20, 332–342. 10.1016/j.molmed.2014.02.007 24667139 PMC5500204

[B18] Osman M. Benamrouz S. Guyot K. Baydoun M. Frealle E. Chabe M.. (2018). High association of *Cryptosporidium* spp. infection with colon adenocarcinoma in lebanese patients. PLoS ONE 12:e0189422. 10.1371/journal.pone.0189422 29261714 PMC5736188

[B19] Pan L. Milligan L. Michaeli J. Cesarman E. Knowles D. M. (2001). Polymerase chain reaction detection of Kaposi's sarcoma-associated herpesvirus-optimized protocols and their application to myeloma. J. Mol. Diagn. 3, 32–38. 10.1016/S1525-1578(10)60647-2 11227070 PMC1907348

[B20] Pavlásek I. (1994). [Localization of endogenous developmental stages of *Cryptosporidium meleagridis* Slavin, 1955 (*Apicomplexa: Cryptosporidiidae*) in birds]. Vet. Med. (Praha). 39, 733–742. 7863574

[B21] Silverlås C. Mattsson J. G. Insulander M. Lebbad M. (2012). Zoonotic transmission of *Cryptosporidium meleagridis* on an organic swedish farm. Int. J. Parasitol. 42, 963–967. 10.1016/j.ijpara.2012.08.008 23022616

[B22] Subramaniam R. Mizoguchi A. Mizoguchi E. (2016). Mechanistic roles of epithelial and immune cell signaling during the development of colitis-associated cancer. Cancer Res. Front. 2, 1–21. 10.17980/2016.1 27110580 PMC4841680

[B23] Sulzyc-Bielicka V. Kołodziejczyk L. Jaczewska S. Bielicki D. Safranow K. Bielicki P.. (2018). Colorectal cancer and *Cryptosporidium* spp. infection. PLoS ONE 13:e0195834. 10.1371/journal.pone.0195834 29672572 PMC5908144

[B24] van Tong H. Brindley P. J. Meyer C. G. Velavan T. P. (2017). Parasite infection, carcinogenesis and human malignancy. EBioMedicine 15, 12–23. 10.1016/j.ebiom.2016.11.034 27956028 PMC5233816

[B25] Wang L. Zhang H. Zhao X. Zhang L. Zhang G. Guo M.. (2013). Zoonotic *Cryptosporidium* species and *Enterocytozoon bieneusi* genotypes in HIV-positive patients on antiretroviral therapy. J. Clin. Microbiol. 51, 557–563. 10.1128/JCM.02758-12 23224097 PMC3553929

[B26] Wesołowska M. Szostakowska B. Kicia M. Sak B. Kvac M. Knysz B. (2016). *Cryptosporidium meleagridis* infection: the first report in Poland of its occurrence in an HIV-positive woman. *Ann*. Parasitol. 62, 239–241. 10.17420/ap6203.58 27770764

[B27] Wolska-Kusnierz B. Bajer A. Caccio S. Heropolitanska-Pliszka E. Bernatowska E. Socha P.. (2007). *Cryptosporidium* infection in patients with primary immunodeficiencies. J. Pediatr. Gastroenterol. Nutr. 45, 458–464. 10.1097/MPG.0b013e318054b09b 18030213

[B28] Xiao L. (2010). Molecular epidemiology of cryptosporidiosis: an update. Exp. Parasitol. 124, 80–89. 10.1016/j.exppara.2009.03.018 19358845

[B29] Xiao L. Escalante L. Yang C. Sulaiman I. Escalante A. A. Montali R. J.. (1999). Phylogenetic analysis of *Cryptosporidium* parasites based on the small-subunit rRNA gene locus. Appl. Environ. Microbiol. 65, 1578–1583. 10103253 10.1128/aem.65.4.1578-1583.1999 PMC91223

